# Randomized controlled pilot study: effect of carrageenan emulsifier on inflammation and gastrointestinal symptoms in quiescent ulcerative colitis

**DOI:** 10.29219/fnr.v67.9575

**Published:** 2023-10-30

**Authors:** Reijo Laatikainen, Markku Lehto, Noora Mäkelä-Salmi, Markku Hillilä, Per-Henrik Groop, Hanne Salmenkari

**Affiliations:** 1 Booston Oy Ltd, Helsinki, Finland; 2 Folkhälsan Institute of Genetics, Folkhälsan Research Center, Helsinki, Finland; 3 Department of Food and Nutrition, University of Helsinki, Helsinki, Finland; 4 Clinic of Gastroenterology, University of Helsinki and Helsinki University, Hospital Jorvi, Espoo, Finland; 5 Abdominal Center, Nephrology, University of Helsinki and Helsinki University Hospital, Helsinki, Finland; 6 Research Program for Clinical and Molecular Metabolism, Faculty of Medicine, University of Helsinki, Helsinki, Finland; 7 Department of Diabetes, Central Clinical School, Monash University, Melbourne, Australia

**Keywords:** carrageenan, ulcerative colitis, IBD, inflammatory bowel disease, emulsifier

## Abstract

**Background:**

Animal models have provided some evidence of the pro-inflammatory effects of the commonly used emulsifier carrageenan. However, the effects of food-grade carrageenan among people with ulcerative colitis (UC) are largely unknown.

**Methods:**

A randomized, placebo-controlled cross-over study comparing high molecular carrageenan and oat-based beta-glucan preparation (placebo) among patients (*n* = 7) with quiescent UC was performed. Primary endpoint was Simple Clinical Colitis Activity Index (SCCAI) at the end of the treatment (7th day). Secondary analyses included biochemical biomarkers of inflammation, intestinal permeability, detoxification of intestinal lipopolysaccharide (LPS), and gastrointestinal symptoms measured by visual analog scale.

**Results:**

There were no statistically significant differences in SCCAI or any biochemical markers between carrageenan and placebo periods, nor were there any significant differences when comparing either period to baseline. Gastrointestinal symptoms were higher during the placebo period; the sum of all symptoms and borborygmi was statistically significantly higher at the end of the placebo period than at the end of the carrageenan period (20.8 ± 18.6 vs. 13.3 ± 16.4; *P* = 0.031, and 29.7 ± 28.6 vs. 17.9 ± 23.6; *P* = 0.016).

**Conclusions:**

Our study suggests that at least short-term usage of food-grade carrageenan is safe among people with UC, but given the limitations of the current study, robust human studies are still urgently needed.

## Popular scientific summary

- Carrageenan is commonly used food additive. It may be found in some milk-based products, dairy alternatives, processed meats and ready-made meals.
- Pre-clinical data has shown that carrageenan might have pro-inflammatory effects in gut, especially worsening or triggering inflammatory bowel disease, such as ulcerative colitis.
- In our randomized trial the effects of food-grade carrageenan were neutral in this vulnerable patient group suggesting short-term usage of food-grade carrageenan is safe.

The incidence of inflammatory bowel disease (IBD) is increasing, affecting approximately 0.7% of different populations in Western countries. Increase in the incidence of IBD has been particularly high in Finland and Nordic countries (1). Dietary factors, along with other environmental factors, are postulated to play a role in this development (2). Increased consumption of processed foods, low intake of fiber, and overconsumption of red and processed meat represent typical major shifts in dietary practices in many Western populations (3). In recent clinical studies, the reduction of neither red meat consumption nor fiber supplements has shown promise in the prevention of colitis in people with IBD (4, 5). The question of whether food processing, especially food additives, plays a crucial role in the development of colitis, is largely unknown – therefore, there is an urgent need for randomized studies in this area of research (6).

Carrageenan is a red seaweed-based emulsifier broadly used by food industry (7). It is a sulfated polygalactan. High molecular weight carrageenan (>200,000 daltons) is used as food-grade carrageenan. It is commonly used in puddings, milk, soy- and oat-based drinks/creams, yogurts, low-fat ice creams, jellies, and processed meat (7). The intake of carrageenan in European Union is estimated to be on average 5.0–88.9 mg/kg (body weight) per day in adults according to European Food Safety Association (EFSA) (8). In populations, which use very little milk-based processed foods, mean carrageenan consumption can be as low as 5 mg/kg. Based on the recent large (*n* = 106,000) French cohort study, carrageenan was the third most consumed food additive, among 90 additives included in the evaluation (9).

In contrast to the high molecular weight (>200,000 Da) carrageenan, the very low molecular weight carrageenan, also called as poligeenan (<20,000 Da), is used to cause experimental colitis in animal models of ulcerative colitis (UC) (10). In a preclinical study, high molecular weight carrageenan was not fermented by human intestinal microbiome, but low molecular weight carrageenan was, and it also caused inflammation via this fermentation cascade (11). Given the pro-inflammatory effects of low molecular weight carrageenan, it cannot be used as a food additive in the European Union (8).

One small (*n* = 5+7) parallel group study suggested that food-grade carrageenan might be pro-inflammatory among people with UC (12). In this study, Simple Clinical Colitis Activity Index (SCCAI) was slightly higher during the carrageenan period versus placebo period. These preliminary results have not been confirmed by other research groups. Therefore, effects of food-grade carrageenan on gastrointestinal symptoms, inflammatory, and permeability markers in people with UC are largely unknown, and more placebo-controlled randomized controlled trials are urgently warranted (13).

The aim of this study was to explore if food-grade carrageenan causes gastrointestinal symptoms, inflammation, and hyperpermeability in patients with UC when consumed at high normal level.

## Materials and methods

Subjects aged 18–64 years were recruited via advertisements in Facebook and the internet (www.pronutritionis.net and www.tervevatsa.fi). The main inclusion criterion was previously diagnosed quiescent UC. Quiescent UC was defined by all of the following: physician global assessment, stable medications, no IBD flare in the previous 3 months, negative result in the Actim^®^ calprotectin rapid test, and clinician- and patient-based UC activity rated as inactive (SCCAI score ≤ 5).

Patients were treated by either lifestyle, 5-ASA, or thiopurine derivatives such as azathioprine. Further inclusion criteria included willingness to consume carrageenan and beta-glucan product, that is, oat fiber (placebo) for 7-day treatment periods. Subjects were excluded if they had relapse (i.e. active disease phase) of UC, use of biologic treatment modalities, or oral corticosteroids for a relapse, intestinal surgery, stoma, cancer, or other severe illness, which might affect patients’ ability to participate in this study. Also, pregnant and lactating women, subjects taking medications potentially influencing gastrointestinal function, and subjects participating in any other clinical trial were not eligible.

The sample size calculation was based on the primary outcome measure SCCAI (14). Suitable published data were not available to be used in power calculations. Therefore, we assumed that the difference between study products to be at least 2 points on the 19-point SCCAI score, and that the standard deviation of that difference would be 1.5 points. Thus, a sample size of 14 would have 90% power to detect this 2-point difference when using a paired t-test with a 0.05 two-sided significance level. The anticipated drop out was 10–20%, and therefore, 16 patients were targeted for this cross-over study.

Prescreening of candidates was done in a telephone interview. Subjects meeting preliminary inclusion criteria were invited to a screening study visit, where their health status, disease activity (remission/relapse), possible medications, and dietary restrictions were evaluated. Before entering the study, all subjects were provided a written informed consent. The study protocol was approved by the ethics committee of the Hospital District of Helsinki and Uusimaa (Approval Code: HUS/770/2021; 7th of April, 2021). The trial was registered at ISRCTN registry, BioMed Central Limited, London, United Kingdom, with a registration code ISRCTN90354393.

### Withdrawal from the study

Participants had the right to withdraw from the study at any time without an obligation to give reasons for the discontinuation. Information about right to withdraw was given to all participants before entering the study both verbally and in written form.

### Screening visit

All eligible participants were asked to participate in the screening visit. During the visit, all possible diseases and medications, height, weight, and sex were enquired using a survey. The nature and the course of the study were explained in detail. The eligible participants were informed via email, and a first research visit was agreed upon.

### Course of the study

**Fig. 1:**
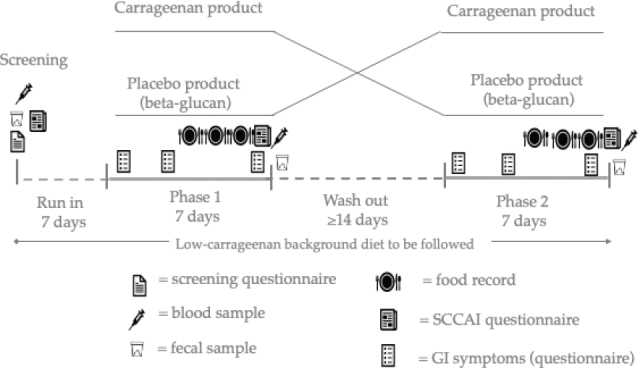
Study design.

This was a randomized cross-over study where each participant consumed 7 days of both carrageenan and placebo (oat fiber, i.e. beta-glucan-rich product). A wash out period of minimum 14 days separated the periods from each other. Randomization was performed in blocks of four by an automated program. A person who was not involved with enrolling the participants or assigning them to the interventions generated the randomization list. Both the investigators and the subjects were blinded to the randomization until the results were analyzed. The study products were given to the participants at the beginning of each treatment period. Furthermore, all participants were instructed to follow an otherwise carrageenan-free diet by the study dietician. The study design is illustrated in Fig. 1 and the flow chart in the Supplementary File 1.

### Research visits

All participants visited the research center at the beginning of each period, that is three times in total. Visits were organized at Selexlab Oy, Helsinki. Blood samples were taken on each visit, SCCAI questions were interviewed by the study dietician or nurse, and the use of VAS symptom questionnaires was instructed and delivered to the participants. Fecal samples were taken at home or in the lab, on the morning of the visit day, and given to researchers when checking into the visit.

### Disease activity, symptom assessment, and food diaries

SCCAI Score (Simple Clinical Colitis Activity Instrument) was used as the primary outcome, which is a validated questionnaire to assess the activity of IBD during the last 7 days (11, 12); the questionnaire was filled by the research dietician who interviewed the participants covering all the SCCAI questions. The study dietician remained blinded for whether the participants had been on carrageenan or placebo when SCCAIs were done. The Finnish version of SCCAI questionnaire is included in the Supplementary File 2.

A total of 10 gastrointestinal symptoms were recorded on a visual analog scale (VAS 0–100 mm for each) at the baseline (screening visit) and at days 1, 3, and 7 of both treatment periods. Food diaries were filled for 3 last days during the two treatment periods.

### Blood, urine, and fecal samples

Blood samples (20 mL) were drawn from antecubital veins, after overnight fast, three times as depicted in Fig. 1. High Sensitivity C-Reactive Protein (Hs-CRP) (inflammatory marker) and creatinine were analyzed by Selexlab Oy, and FABP-2 (enterocyte damage marker) was analyzed using Human FABP2/I-FABP DuoSet ELISA (R&D Systems, Minnesota, MO, USA). Calprotectin was analyzed by using the Bühlmann method (15).

Fecal samples were taken at home from the first gut passage of the day to a fecal collection tube and brought to the appointment and frozen on the same day. Fecal calprotectin (inflammatory marker) and fecal intestinal alkaline phosphatase (IAP) activity were determined from the fecal samples. Overnight urine collections were done at the same timepoints as fecal sample collection and used for albumin and creatinine determinations.

IAP activity was measured using an inhouse method as described earlier (16). Briefly, fecal samples were vortexed for 2 min in extraction buffer (10 mM Tris-HCl, 1 mM MgCl_2_, 0.1 mM ZnCl_2_, pH 8) containing cOmplete™, EDTA-free Protease Inhibitor Cocktail (Roche, Mannheim, Germany). After a 30-min-incubation at +4°C, the samples were centrifuged at 15,700 g at +4°C for 10 min, and the supernatant was used for IAP activity and protein concentration assays. For IAP activity assay, known amounts of IAP substrate, p-nitrophenyl phosphate (pNPP, Sigma-Aldrich, St. Louis, MO, USA), were treated with a constant amount of calf IAP (Sigma-Aldrich) in assay buffer (10 mM Tris-HCl, 1 mM MgCl_2_, 0.1 mM ZnCl_2_, pH 10) to create a p-nitrophenol (pNP) standard curve. Samples with unknown alkaline phosphatase activity were treated with excess (4.56 mM) pNPP. The standards and samples were incubated at +37°C for 30 min, after which the reactions were stopped by the addition of 3M NaOH, and absorbance was read at 405 nm, the absorption maximum of pNP. IAP activity in units per mL was calculated as the production of pNP in μmol*mL-1*min-1. IAP activity was normalized to the corresponding protein concentration of the samples assayed with Pierce™ BCA Protein Assay Kit (Thermo Fisher Scientific, Waltham, MA, USA).

### Background diet

All participants were instructed to follow carrageenan-free diet. In practice, puddings, flavored yogurts and oat/soy yogurts, whipped creams, long self-life creams, oat crème fraiche, milk shakes, vanilla and chocolate sauces, low-fat ice creams, convenience foods, marmalades, sausages, and cold cuts were avoided during all study periods, including run-in and washout periods, unless verified by the study dietician that they were carrageenan free. The participants were instructed to observe and avoid carrageenan in all prepackaged products they bought and used. The study dietician instructed carrageenan-free diet to all participants according to the preformed list, Supplementary File 3 (in Finnish). Participants were encouraged to maintain their current level of exercise and sleeping habits. Heavy drinking, more than two standard doses of alcohol/day, was discouraged throughout the study.

### Carrageenan and placebo products

Food-grade carrageenan (Genulacta^®^ LP-60, Azelis Oy, Finland), with a molecular weight of 400,000–550,000 Da, was provided to the participants as powder to be dissolved with heated oat-based drink (Oddly Good Barista, Valio Oyj, Finland). Oat drink-based chocolate shake was prepared at homes of each participant, so that 2 dL of the oat drink contained 1,000 mg carrageenan and the needed amount of sucrose-sweetened chocolate powder (20 g/day). Each participant needed to drink 4.0 dL of the shake/day to achieve targeted intake (2,000 mg) of carrageenan solely from the study product. This corresponds 29 mg/kg for an adult weighing 70 kg, which is well within the regular level of consumption in EU [8]. Placebo drink was composed in a similar manner, and the only difference is that carrageenan was replaced by oat fiber, high in beta-glucan (Alku Voimakaura, runsaskuituinen, Fazer ltd). The amount of beta-glucan was calculated similar to carrageenan, that is, 2,000 mg/day. Oat, a gluten-free grain, is usually well tolerated by the patients with IBD and other gastrointestinal diseases/disorders (17). Furthermore, oat fiber is shown to increase colonic butyrate formation in UC (18), thus possibly possessing some anti-inflammatory features. Oat fiber (beta-glucan) also forms gel-like viscosity when heated and mixed with water, thus resembles carrageenan from the physio-rheological properties; consequently, oat fiber preparation was reasonably well suited for serving as placebo. Both products were packed together with cocoa powder into small identical transparent plastic pouches (Supplementary File 4), and oat drink was provided for free.

### Statistical analysis

VAS scores at the end of each treatment and SCCAI scores were compared using a two-tailed Wilcoxon matched-pairs signed rank test. The results of laboratory analyses were analyzed using repeated measures one-way ANOVA, except for albumin, which was analyzed using the Friedman test. Log10 transformed values were used for Calprotectin, FABP2, and IAP. The statistical testing was analyzed using GraphPad Prism version 9 (San Diego, CA, USA).

## Results

### Characteristics of subjects

Due to COVID-19 and the negative reputation of carrageenan among the potential participants, the recruitment was very challenging in Helsinki. We were able to recruit 7 participants during 2021–2022. The acclaimed negative health effects of carrageenan were highlighted by social media discussants in the recruitment channels, and a participation into any carrageenan studies was overtly discouraged.

All 7 participants were females (100%). Their median age was 45 (range 38–60) years, and the median body mass index was 27.4 (range 20.3–37.7) kg/m^2^. Other baseline characteristics including laboratory values are depicted in Table 1. SCCAI scores ranged from 0 to 3; thus, all participants had their disease in quiescent phase, that is SCCAI scores were <5 (scale 0–20). All participants also had negative result in rapid calprotectin test, and the clinical evaluation performed by the gastroenterologist supported these findings.

**Table 1:** Baseline characteristics of the participants, mean values (range)

	All, *n* = 7
Age, years	*45* (*38*–6*0*)
BMI, kg/m^2^	2*7*.*4* (*20.3*–37.7)
SCCAI (scale 0–19)	1.7 (0–3)
HS-CRP* mg/L	1.4 (0.6–2.8)
F-Calpro (μg/g)	333 (30–940)
F-FABP-2 (pg/mL)	449 (228–1,066)
F-IAP (units/g)	365 (33–1,886)
U-Crea (mmol/L)	8.0 (4–13.3)
FS-Crea (μmol/L)	62.4 (48–73)
U-Alb** (mg/L)	3 (3.0–3.0)

* Values under 0.6 were undetectable by the quantification method.

** Values under 3.0 were undetectable by the quantification method.

### Inflammatory and other biochemical markers

We did not find any statistically significant differences between the treatment periods (baseline, carrageenan, and placebo) in any biochemical markers. The outcomes are presented in Table 2.

**Table 2:** Results of biochemical measurements at baseline (day 0) and at the end of each 7-day period. Results are given as average and as standard deviations (±), (*n* = 7)

	Baseline (B)	Carrageenan (0)	Placebo (00)	*P*-value B versus 0*	*P*-value B versus 00*	*P*-value 0 versus 00*
F-Calpro (μg/g)	333 (± 346)	287 (± 408)	241 (± 223)	0.8511	0.8511	0.8511
S-FABP2 (pg/mL)	449 (± 264)	466 (± 172)	506 (± 156)	0.8358	0.227	0.6273
F-IAP (units/g)	365 (± 624)	552 (± 774)	379 (± 657)	0.2973	0.8298	0.2477
HS-CRP (mg/L)	1.4 (± 0.9)	2.5 (± 1.3)	1.6 (± 1.1)	0.1861	0.4064	0.3018
U-Crea (mmol/L)	8.0 (± 3.7)	7.9 (± 5.1)	7.9 (± 4.9)	0.9991	0.9985	>0.9999
FS-Crea (μmol/L)	62 (± 11)	64 (± 12)	64 (± 13)	0.5587	0.8393	0.9967
U-Alb (mg/L)	3.0 (± 0.0)	4.7 (± 4.2)	4.3 (± 3.1)	>0.9999	>0.9999	>0.9999

* p-values; Repeated Measures one-way ANOVA.

### Simple Clinical Index and gastrointestinal symptoms

The primary endpoint in this study was SCCAI at the end of the treatment periods; there was no difference in the SCCAI between carrageenan and placebo periods 1.0 (1.0, SD) versus 2.0 (2.0) (*P* = 0.250). We did not find any statistically significant difference in SCCAI when either treatment period was compared to the baseline.

We observed a statistically significant effect in the gastrointestinal symptom score between the carrageenan and the placebo periods; sum of symptoms at the end of the treatment was lower during the carrageenan period when compared to the placebo period 13.3 (± 16.4) versus 20.8 (± 18.6) (*P* = 0.031). The absolute values for individual symptoms tended to be higher during the placebo period than during the carrageenan period, but only the difference in borborygmi was statistically significant. The results of clinical endpoints at the end of the periods are presented in Table 3.

**Table 3:** Assessment of symptoms (SCCAI and VAS-Based) at the end of each 7-day period. Results are given as average ± standard deviations (*n* = 7)

	Carrageenan	Placebo	*P*
**SCCAI score**	**1.0 (1.0)**	**2.0 (2.0)**	**0.250**
Sum Score 12 symptoms (VAS)*	**13.3 ± 16.4**	**20.8 ± 18.6**	**0.031**
Bloating	22.0 ± 28.7	42.6 ± 30.7	0.063
Flatulence	32.4 ± 27.8	41.9 ± 25.8	0.375
Diarrhea	12.9 ± 19.5	13.1 ± 20.7	0.875
Constipation	0.6 ± 1.4	11.0 ± 17.3	0.500
Abdominal pain	12.9 ± 20.4	19.3 ± 33.5	0.500
Cramping	8.7 ± 19.0	0.6 ± 1.4	0.500
Borborygmi	17.9 ± 23.6	29.7 ± 28.6	0.016
Heart burn	0.9 ± 1.5	11.9 ± 20.7	0.500
Dyspepsia	17.1 ± 23.5	21.0 ± 29.2	0.625
Feeling of incomplete defecation	15.7 ± 24.8	24.3 ± 28.8	0.313
Urgent need for defecation	17.6 ± 21.2	23.4 ± 33.2	0.438
Nausea	1.4 ± 2.3	10.7 ± 21.1	0.250

* sum all 12 symptoms on 100 mm VAS/12.

**Fig. 2:**
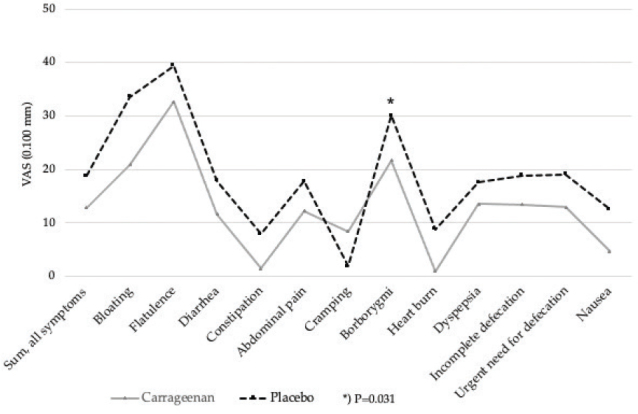
Average values for gastrointestinal symptoms (sum of days 1, 3, and 7), (*n* = 7).

We also analyzed the average (sum of days 1, 3, and 7) gastrointestinal symptoms scores for the entire treatment periods (Fig. 2). Only difference that we found was the occurrence of borborygmi, which was more pronounced during the placebo period 29.7 (± 28.6) versus 17.9 (± 25.8) (*P* = 0.016).

### Dietary and carrageenan measures

Six participants reported the consumption of all pouches during both carrageenan and placebo periods. One participant disclosed to have dismissed one pouch during the placebo period. All participants confirmed to have adhered to low-carrageenan diet with their best possible ability throughout the total study period.

Dietary intakes are reported in the Supplementary File 5. There were no statistically significant differences in the intake of either fiber, carbohydrate, fat, protein, or energy.

## Discussion

We found no proof of pro-inflammatory effect of food-grade carrageenan (molecular weight 400,000–560,000 Da) in our study when compared to placebo (oat fiber) in people with quiescent UC. The primary outcome of this study, SCCAI, was not statistically different neither between carrageenan and placebo periods nor when baseline situation was compared to either carrageenan or placebo. None of the participants experienced a clinical relapse of the disease neither during the carrageenan nor during the beta-glucan period. Furthermore, none of the biochemical inflammatory markers was elevated during the carrageenan period when compared to either beta-glucan or baseline. On the contrary, gastrointestinal symptoms were experienced slightly more often during the placebo period versus carrageenan period.

These results contrast with the clinical study published previously, in which carrageenan induced more pro-inflammatory reactions than placebo (dextrose) in people with UC (12). Many features differed between the studies. The current carrageenan dose per day (2,000 mg) is a regular European dose per day (8), exceeding 10 times the dosage used in the previous study (200 mg/day). The previous study was a long-term study, while our study lasted only a week. In addition, the carrageenan was mixed with oat drink and cocoa in our study, whereas in the previous study, carrageenan was served as capsules. We used beta-glucan preparation as placebo due to its chemical resemblance and favorable clinical outcomes in previous studies (17), whereas dextrose was used in the previous study (12). In line with our study, the previous study did not find statistically significant difference in inflammatory markers between carrageenan and placebo groups, albeit there was statistically significant difference in the SCCAI score (12).

The reason for the discrepancy in the results of these two studies is unknown. Both studies were small; this might be part of the explanation as neither study is robust by its design. It is possible that 7 days used in our study is too short to detect clinical or pro-inflammatory effects in people with UC. It is also possible that capsules, as used in the previous study, may not mimic well enough naturalistic administration of carrageenan as dispersed into food (our study).

It was unexpected that placebo caused more gastrointestinal symptoms, albeit it had no effect on inflammatory values. We chose oat fiber concentrate as placebo because oat was well tolerated in the previous studies among people with UC and other GI disorders (17) and because oat is relatively low in FODMAPs (Fermentable Oligo-, Di-, Mono-saccharides, And Polyols) (19) – common cause of functional gastrointestinal symptoms. It is possible that oat products cause more colonic gas than previously understood (20); however, these findings need to be replicated in further studies. We did not use hydrogen breath tests to quantify gas building in our study; therefore, the reason for slightly worse symptom profile for oat beta-glucan is not known. New studies on the gastrointestinal tolerance of oat products are needed to either confirm, or refute, the recent findings.

Our study could not replicate the findings from animal studies, which have shown clear pro-inflammatory potential of carrageenan (11). Given the results of the current study and the broad coverage of animal studies and relatively strong dietary recommendations based on these animal models (21), new robust human studies are urgently needed. In 2021, a feasibility study on the diet low in food additives, and its effect on Crohn’s disease, was executed (22), but apart from ours and the aforementioned study (12), no other randomized controlled studies have been performed with food additives and their effects on inflammatory markers and clinical activity of these diseases, as far as we know.

Our study has some strengths and weaknesses. Adherence to treatments was excellent, and we used established (CRP, calprotectin, and FABP-2) and novel markers (f-IAP). Randomized, matched placebo-controlled design is a further strength. Calprotectin was measured by the Bühlmann’s method, which yields at least 2-fold higher values than other methods (15) – this explains, at least partly, the relatively high baseline values of calprotectin. All patients were clinically in the remission at baseline as assessed by SCCAI, the gastroenterologist, blood tests, and the Actim rapid test. Major weaknesses of this study are the relatively short duration and the serious difficulties in recruiting participants, forcing us to leave the final participant count lower than what we aimed at. During the planning period of this study, it was not possible to estimate how strong and quick might be the potential inflammatory effect of carrageenan in our human population. We were somewhat concerned of triggering relapses of the disease, based on the findings of the preclinical studies. This was the main reason for choosing the short study period. In retrospect, we might not have been able to gather even these many patients for a longer study. Nevertheless, all participants showed excellent adherence to the procedures. The small group size was partly mitigated by the fact that participants served as their own control (cross-over study), allowing *n* = 7 for both periods. We cannot totally rule out carry-over effect for GI symptoms because we did not do baseline VAS assessments, but for obvious reasons, the carry-over effect is not an issue for inflammatory outcomes; there was no change neither in any inflammatory markers nor in any drop-outs. In the light of these facts, the results of new randomized trials in UC or Crohn’s disease including more participants are very much awaited.

## Conclusions

We found that food-grade carrageenan with large molecular weight does not cause pro-inflammatory changes or gastrointestinal symptoms in people with quiescent UC. Our study suggests that at least short-term usage of carrageenan is safe among people with UC, but given the duration and size of the current study, new larger studies are urgently needed. Our study also shows that carrageenan supplementation in conjunction with low-carrageenan diet is feasible to implement even if the recruitment of the participants with UC may be very challenging.

## References

[1] Jussila A, Virta LJ, Salomaa V, Mäki J, Jula A, Färkkilä MA. High and increasing prevalence of inflammatory bowel disease in Finland with a clear North-South difference. J Crohn’s Colitis 2013; 7(7): e256–62. doi: 10.1016/j.crohns.2013.01.001 23140840

[2] Levine A, Sigall Boneh R, Wine E. Evolving role of diet in the pathogenesis and treatment of inflammatory bowel diseases. Gut 2018; 67(9): 1726–38. doi: 10.1136/gutjnl-2017-314968 29777041

[3] Baker P, Machado P, Santos T, Sievert K, Backholer K, Hadjikakou M, et al. Ultra-processed foods and the nutrition transition: global, regional and national trends, food systems transformations and political economy drivers. Obes Rev 2020; 21(12): e13126. doi: 10.1111/obr.13126 32761763

[4] Albenberg L, Brensinger CM, Wu Q, Gilroy E, Kappelman MD, Sandler RS, et al. A diet low in red and processed meat does not reduce rate of Crohn’s disease flares. Gastroenterology 2019; 157(1): 128–36.e5. doi: 10.1053/j.gastro.2019.03.043 30872105 PMC6726378

[5] Wedlake L, Slack N, Andreyev HJ, Whelan K. Fiber in the treatment and maintenance of inflammatory bowel disease: a systematic review of randomized controlled trials. Inflamm Bowel Dis 2014; 20(3): 576–586. doi: 10.1097/01.MIB.0000440919.02606.f1 24445775

[6] Marion-Letellier R, Amamou A, Savoye G, Ghosh S. Inflammatory bowel diseases and food additives: to add fuel on the flames! Nutrients 2019; 11(5): 1111. doi: 10.3390/nu11051111 31109097 PMC6567822

[7] McKim JM. Food additive carrageenan: Part I: a critical review of carrageenan in vitro studies, potential pitfalls, and implications for human health and safety. Crit Rev Toxicol 2014; 44(3): 211–43. doi: 10.3109/10408444.2013.859292 24456237

[8] EFSA Panel on Food Additives and Nutrient Sources added to Food (ANS). Re-evaluation of carrageenan (E 407) and processed Eucheuma seaweed (E 407a) as food additives. EFSA J 2018; 16(4): e05238. doi: 10.2903/j.efsa.2018.5238 32625873 PMC7009739

[9] Chazelas E, Druesne-Pecollo N, Esseddik Y, De Edelenyi FS, Agaesse C, De Sa A, et al. Exposure to food additive mixtures in 106,000 French adults from the NutriNet-Santé cohort. Sci Rep 2021; 11(1): 19680. 34608173 10.1038/s41598-021-98496-6 PMC8490357

[10] Watt J, Marcus R. Experimental ulcerative disease of the colon in animals. Gut 1973; 14(6): 506–510. doi: 10.1136/gut.14.6.506 4581171 PMC1412737

[11] Yin Y, Li M, Gu W, Zeng B, Liu W, Zhu L, et al. Carrageenan oligosaccharides and associated carrageenan-degrading bacteria induce intestinal inflammation in germ-free mice. J Genet Genomics 2021; 48(9): 815–24. doi: 10.1016/j.jgg.2021.07.002 34400364 PMC8628850

[12] Bhattacharyya S, Shumard T, Xie H, Dodda A, Varady KA, Feferman L, et al. A randomized trial of the effects of the no-carrageenan diet on ulcerative colitis disease activity. Nutr Healthy Aging 2017; 4(2): 181–92. doi: 10.3233/NHA-170024 28447072 PMC5389019

[13] Bancil AS, Sandall AM, Rossi M, Chassaing B, Lindsay JO, Whelan K. Food additive emulsifiers and their impact on gut microbiome, permeability, and inflammation: mechanistic insights in inflammatory bowel disease. J Crohns Colitis 2021; 15(6): 1068–79. doi: 10.1093/ecco-jcc/jjab036 33336247

[14] Walmsley RS, Ayres RC, Pounder RE, Allan RN. A simple clinical colitis activity index. Gut 1998; 43(1): 29–32. doi: 10.1136/gut.43.1.29 9771402 PMC1727189

[15] Lin L, Wyness SP, Jensen R, Bird J, Norgyal T, Jensen G, et al. Comparison of next-generation assays for fecal calprotectin vs the PhiCal assay. Am J Clin Pathol 2022; 157(2): 252–6. doi: 10.1093/ajcp/aqab017 34390332

[16] Lassenius MI, Fogarty CL, Blaut M, Haimila K, Riittinen L, Paju A, et al. Intestinal alkaline phosphatase at the crossroad of intestinal health and disease – a putative role in type 1 diabetes. J Intern Med 2017; 281(6): 586–600. doi: 10.1111/joim.12613 28393441

[17] Thies F, Masson LF, Boffetta P, Kris-Etherton P. Oats and bowel disease: a systematic literature review. Br J Nutr 2014; 112(suppl 2): S31–S43. doi: 10.1017/S0007114514002269 25267242

[18] Hallert C, Björck I, Nyman M, Pousette A, Grännö C, Svensson H. Increasing fecal butyrate in ulcerative colitis patients by diet: controlled pilot study. Inflamm Bowel Dis 2003; 9(2): 116–21. doi: 10.1097/00054725-200303000-00006 12769445

[19] Ispiryan L, Kuktaite R, Zannini E, Arendt EK. Fundamental study on changes in the FODMAP profile of cereals, pseudo-cereals, and pulses during the malting process. Food Chem 2021; 343: 128549. doi: 10.1016/j.foodchem.2020.128549 33189480

[20] Laito S, Valkonen N, Laaksonen O, Kalliomäki M, Tuure T, Linderborg KM. Effect of oat or rice flour on pulse-induced gastrointestinal symptoms and breath hydrogen in subjects sensitive to pulses and controls – a randomised cross-over trial with two parallel groups. Br J Nutr 2022; 128(11): 2181–92. doi: 10.1017/S0007114522002020 35086570 PMC9661369

[21] Adolph TE, Zhang J. Diet fuelling inflammatory bowel diseases: preclinical and clinical concepts. Gut 2022; 71(12): 2574–86. doi: 10.1136/gutjnl-2022-326272 36113981 PMC9664119

[22] Sandall AM, Cox SR, Lindsay JO, Gewirtz AT, Chassaing B, Rossi M, et al. Emulsifiers impact colonic length in mice and emulsifier restriction is feasible in people with Crohn’s disease. Nutrients 2020; 12(9): 2827. doi: 10.3390/nu12092827 32942699 PMC7551245

