# Supplementary material for: Randomized controlled pilot study: effect of carrageenan emulsifier on inflammation and gastrointestinal symptoms in quiescent ulcerative colitis
Source: Food Nutr Res. 2023 Oct 30;67:10.29219/fnr.v67.9575. doi: 10.29219/fnr.v67.9575 (PMC10619385; doi:10.29219/fnr.v67.9575)
Supplement: Supplementary file 1 [file FNR-67-9575-s001.docx]

**Supplements**

Supplement 1. The Flow chart.


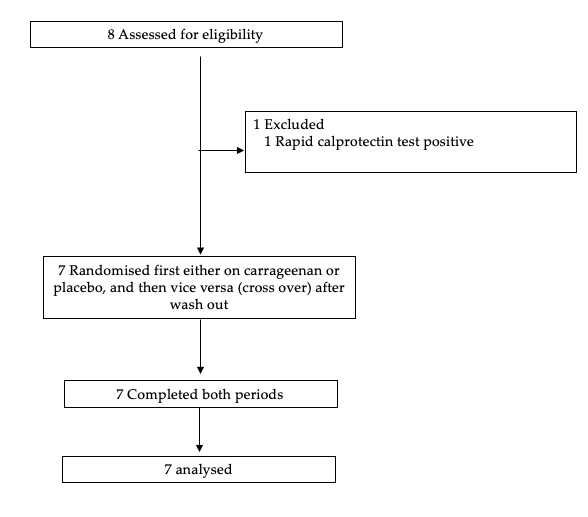


Supplement 2. Simple Clinical Colitis Activity Index, Finnish questionnaire


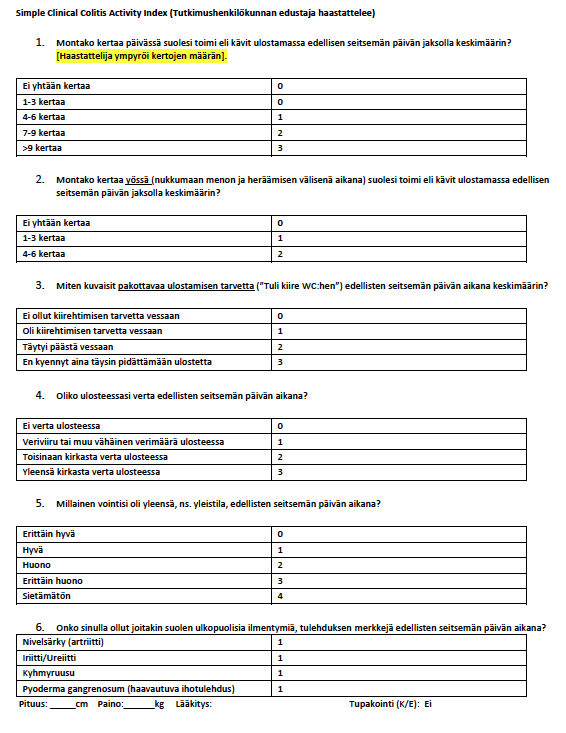


Supplement 3. List of food items containing carrageenan, to be avoided (in Finnish).


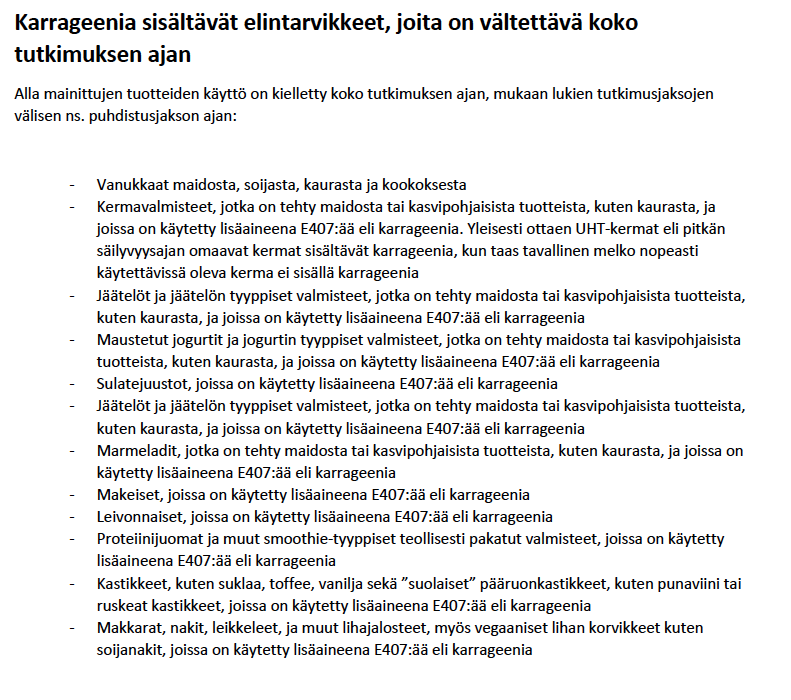


Supplement 4. Pre-packed pouches containing carrageenan (0) and placebo (oat fiber, high in beta-glucan). 0 and 00 samples were provided during different study visits so that the participants were not able to compare packages at the same time.


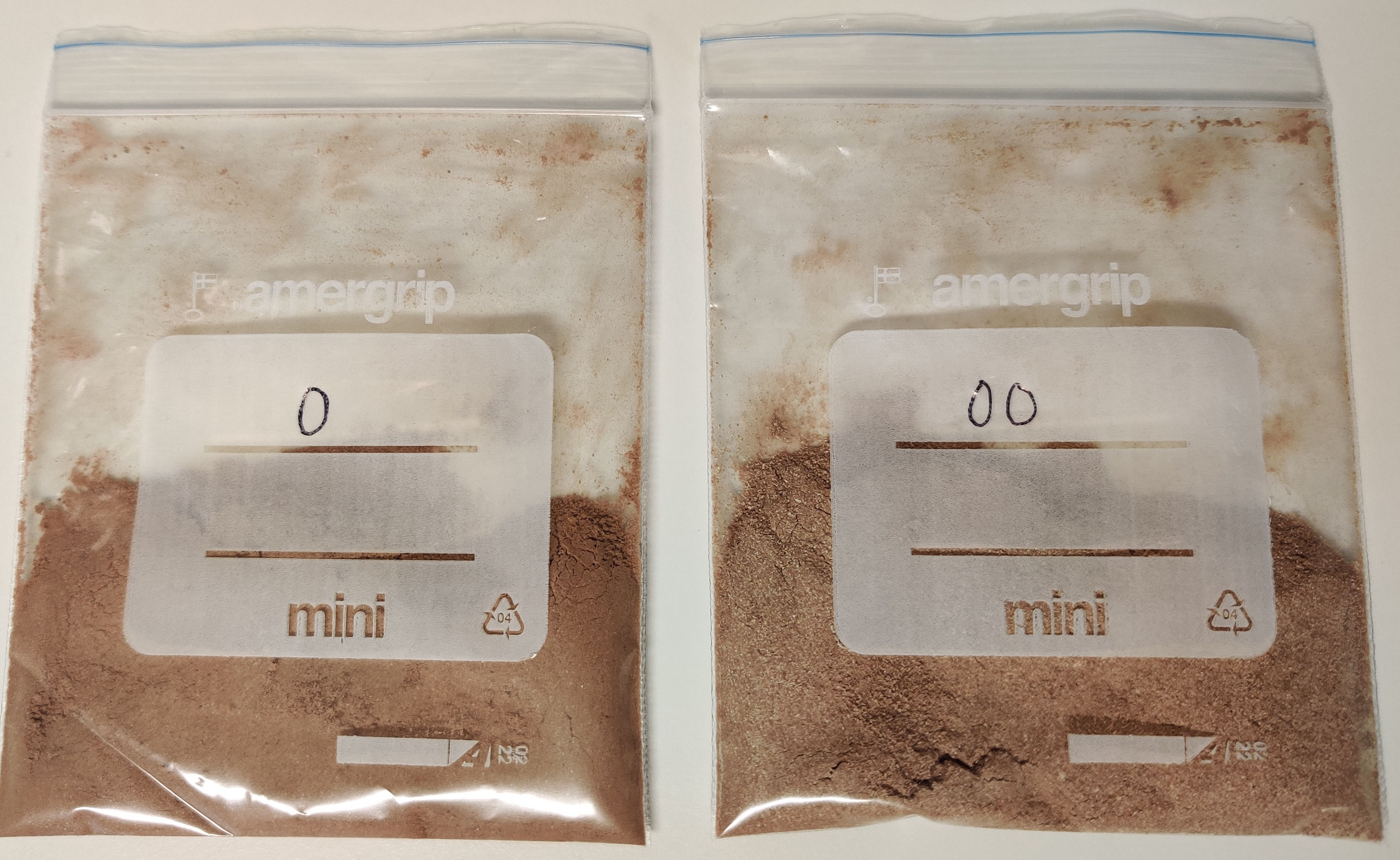


Supplement 5. Dietary intakes during the intervention (n=6).

|  | Placebo (average, sd) | Carrageenan (average, sd) | p-value* |
| --- | --- | --- | --- |
| Energy, Kj | 8412 (1492) | 8519 (1254) | 0.869 |
| Carbohydrate, grams | 207 (39) | 218 (33) | 0.556 |
| Protein, grams | 90 (7.9) | 88 (17) | 0.600 |
| Fat, grams | 83 (20) | 81 (12) | 0.772 |
| Fiber, grams | 24 (5.3) | 24 (6.5) | 0.884 |

*) paired sample t-test for all other nutrients except for protein, in which Wilcoxon signed rank test was used due to nonparametric distribution of protein intakes
